# Emerging two-dimensional ferromagnetism in silicene materials

**DOI:** 10.1038/s41467-018-04012-2

**Published:** 2018-04-26

**Authors:** Andrey M. Tokmachev, Dmitry V. Averyanov, Oleg E. Parfenov, Alexander N. Taldenkov, Igor A. Karateev, Ivan S. Sokolov, Oleg A. Kondratev, Vyacheslav G. Storchak

**Affiliations:** National Research Centre “Kurchatov Institute”, Kurchatov Sq. 1, Moscow, 123182 Russia

## Abstract

The appeal of ultra-compact spintronics drives intense research on magnetism in low-dimensional materials. Recent years have witnessed remarkable progress in engineering two-dimensional (2D) magnetism via defects, edges, adatoms, and magnetic proximity. However, intrinsic 2D ferromagnetism remained elusive until recent discovery of out-of-plane magneto-optical response in Cr-based layers, stimulating the search for 2D magnets with tunable and diverse properties. Here we employ a bottom-up approach to produce layered structures of silicene (a Si counterpart of graphene) functionalized by rare-earth atoms, ranging from the bulk down to one monolayer. We track the evolution from the antiferromagnetism of the bulk to intrinsic 2D in-plane ferromagnetism of ultrathin layers, with its characteristic dependence of the transition temperature on low magnetic fields. The emerging ferromagnetism manifests itself in the electron transport. The discovery of a class of robust 2D magnets, compatible with the mature Si technology, is instrumental for engineering new devices and understanding spin phenomena.

## Abstract (web summary)

Exploring the magnetism in 2D materials paves the way to low-dimensional spintronics. Here the authors report evolution of bulk antiferromagnetism to intrinsic 2D in-plane ferromagnetism in layered structures of silicene functionalized by rare-earth atoms as they are scaled down to one monolayer.

## Introduction

Realization of long-range magnetic order in low-dimensional systems is an intriguing challenge holding promise for future nanoelectronics and spintronics^1,2^. It is a daunting task since the long-range magnetic order is suppressed by thermal fluctuations. This statement is the celebrated Mermin–Wagner theorem^3^, which is valid for spin-rotational invariant low-dimensional systems with exchange interactions. However, both magnetic anisotropy and dipolar interactions are well capable of lifting the Mermin–Wagner restriction. Certainly, there is no lack of attempts to endow two-dimensional (2D) materials with magnetism. The experimental studies are basically limited to extrinsic magnetism arising from chemical dopants and defects^4^, engineering of edges^5^ and effects of proximity to magnetic layers^6,7^. The intrinsic 2D magnetism is a different feat: although a study of magnetism as evolving with the number of layers of the parent van der Waals magnet seems rather straightforward, it faces significant obstacles, not the least due to a much greater sensitivity than that provided by conventional magnetometers required to measure the tiny magnetic moments at the monolayer (ML) level^8^. However, reports of intrinsic magnetism in 2D materials started to emerge: antiferromagnetism (AFM)^9^, ferrimagnetism in a supramolecular lattice^10^ and, most recently, out-of-plane ferromagnetism (FM)^11,12^, all detected by optical techniques and citing different mechanisms. In particular, Huang et al.^12^ show that magnetism in CrI_3_ is layer-dependent: the magnetic state transforms from FM to AFM (due to interlayer coupling) and then back to FM as the film thickness changes from 1 ML to 2 ML and then to 3 ML. In a separate study of a few MLs of Cr_2_Ge_2_Te_6_^11^ the dependence of the transition temperature on low magnetic fields is identified as a telltale sign of 2D magnetism. These developments complement the earlier studies of ultrathin magnetic films but “demand a fresh, contemporary perspective on low-dimensional magnetism”[8].

In the case of invariance under a continuous global rotation of the spins, long-range order implies a gapless branch in the spectrum of spin-wave excitations. In 2D systems with short-range interactions (such as exchange), such excitations lead to a divergence in the spin-wave amplitude at finite *T*, which destroys the long-range order^13^. Depending on the system symmetry, magnetic anisotropy and dipolar interactions result in opening a gap or pseudogap in the spin-wave spectrum^14,15^, which renders spin fluctuations finite and stabilizes long-range magnetic order. In 2D, the effective transition temperature $T_{\mathrm{C}}^ \ast$ between FM-like and paramagnetic-like states depends crucially on the spin-wave excitation (pseudo)gap^15^. The latter can be controlled with low magnetic fields–in ref.^11^, the dependence of $T_{\mathrm{C}}^ \ast$ on magnetic fields is reproduced in the framework of renormalized spin-wave theory within the Hartree–Fock approximation. This behavior is in sharp contrast to the 3D regime where the influence of an external field is rather weak. It does not mean that the exchange interaction *J* is less important in the 2D magnetism–*T*_C_ is proportional to *J* but relatively weak anisotropy and dipolar interactions trigger the ferromagnetism^15^.

The rise of 2D magnetism is expected to have a transformative effect on nanoelectronics. A particularly fascinating direction is the control of properties in atomically thin materials via proximity exchange as in the recent example of the WSe_2_/CrI_3_ heterostructure^16^. The research on intrinsic magnetism in 2D layers is still in its infancy: the first works whet the appetite for materials with robust FM measurable by conventional magnetometers, with different types of magnetic anisotropy, exhibiting useful properties complementing the magnetism, and what is particularly important, compatible with the current semiconductor technology. The design of new 2D magnets would benefit from the use of building blocks predisposed to magnetic phenomena. In particular, buckled 2D-Xenes^17,18^ –honeycomb lattices of group IVA atoms–offer a platform for spin-related phenomena and spintronic applications. Among them, silicene^19,20^, a tunable 2D Dirac material which can be seamlessly integrated with the Si technology, is expected to exhibit a number of exotic properties such as quantum spin Hall effect, quantum anomalous Hall effect, chiral superconductivity, valley polarized quantum Hall effect. Silicene spintronics has become a vast field of theoretical research^20^ with applications exemplified by an ideal spin filter^21^. FM order in silicene sheets is predicted to arise as long as they are asymmetrically functionalized as in partially hydrogenated^22^ and bilayer^23^ silicene. However, the high reactivity of silicene poses considerable difficulties to any experimentation: free-standing silicene is yet to be demonstrated and only one silicene-based device of note is reported to date^24^. Thus, in practice either silicene is directly integrated with a substrate^19,20^ or forms stacks of alternating silicene and metal monolayers, as in CaSi_2_ which is experimentally shown to host Dirac fermions^25^. The borderline case is integration with the Si(111) substrate which is predicted to be possible by the 1 × 1 metal passivation leading to overlayer silicene preserving its Dirac cones^26^. Recently, we managed to produce epitaxial films on Si(111) of multilayer silicene stoichiometrically intercalated with active metals^27,28^, which could serve as parent compounds for 2D layer structures.

In the following we demonstrate the emergence of intrinsic 2D magnetism in silicene-based materials at the ML level. These rare-earth magnets differ from Cr-based materials by their robustness, type of magnetic anisotropy, suppression of FM in parent compounds, enhanced sensitivity to low magnetic fields, compatibility with Si.

## Results

### Material design

**Fig. 1:**
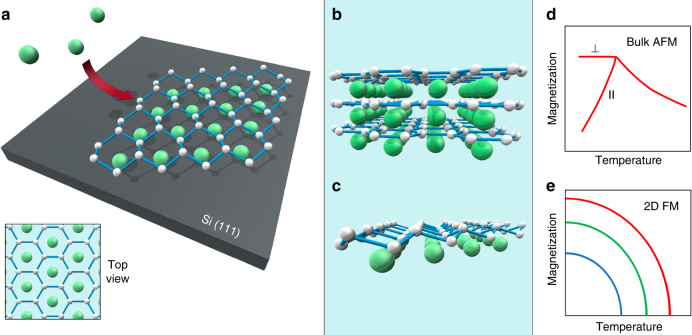
Schematics of the experiment. **a** MBE synthesis of MSi_2_ silicene materials by directing metal atoms (Gd or Eu) at the Si(111) substrate leading to formation of **b** a bulk (multilayer) material or **c** a monolayer. The AFM state of the bulk **d** transforms into 2D ferromagnetism with its characteristic dependence of the transition temperature on magnetic fields **e**

In search of silicene-based 2D magnets, the choice of the metal is important–a robust 2D magnetism is more likely in a system with stable local moments. Here we look for rare-earths, generally known for the strongest FM behavior, and choose the most spin-pure case of Gd^3+^ and Eu^2+^ with a half-filled *f*-shell leading to vanishing orbital (*L* = 0) and maximal spin (*S* = 7/2) angular momenta. The corresponding structures of silicene with Gd and Eu sheets are produced in a highly controlled way by molecular beam epitaxy (MBE). Figure 1a shows a simple scheme of the synthesis: metal atoms (Gd or Eu) are directed at the heated (111) surface of Si. Then, layers of the MSi_2_ stoichiometry are formed with Si atoms delivered to the surface via the well-known vacancy mechanism. This is a leading mechanism of atomic transport (diffusion) in crystalline solids^29^. In the case of silicides, a metal atom at the surface extracts the nearest Si atom from the silicide, thus forming a vacancy. Then, a neighboring Si atom jumps to this vacancy which effectively results in its transport. The vacancy migrates until it reaches the Si substrate. Ultimately, the synthesis results in a multilayer silicene structure–bulk silicide. If unprotected, silicene systems are prone to oxidation by air, as highlighted in ref.^30^. In our study, such unwanted reaction is avoided by using a capping layer of SiO_x_ with a thickness of 200 Å.

Figure 1b shows the structure of GdSi_2_ with planar Si layers^31^, as in graphene. In the case of Eu, the silicene layers are buckled^28^, similar to predictions for free-standing silicene^17^. In fact, buckling is an important parameter governing properties of silicene^32^. However, the synthesis can be terminated early to form one or several MLs of MSi_2_. Silicene sheet is buckled in the ML limit even in GdSi_2_^31^ (Fig. 1c). Bulk GdSi_2_ is known to be AFM. The situation with layered EuSi_2_ is more complex: the compound is pseudomorphic^28^–as long as the film is thicker than 7 nm, EuSi_2_ transforms into its more stable tetragonal polymorph^33^. Magnetization studies of 7 nm films of EuSi_2_ are indicative of coexisting AFM and weak FM behavior^28^. It may suggest transformation of 3D AFM with its characteristic temperature dependence of the magnetic moment (Fig. 1d) into 2D FM in ultrathin films, with its characteristic features like dependence of $T_{\mathrm{C}}^ \ast$ on low magnetic fields (Fig. 1e). Here we test this hypothesis by measuring properties of a series of silicene compounds with different numbers of MLs.

### Structural characterization

**Fig. 2:**
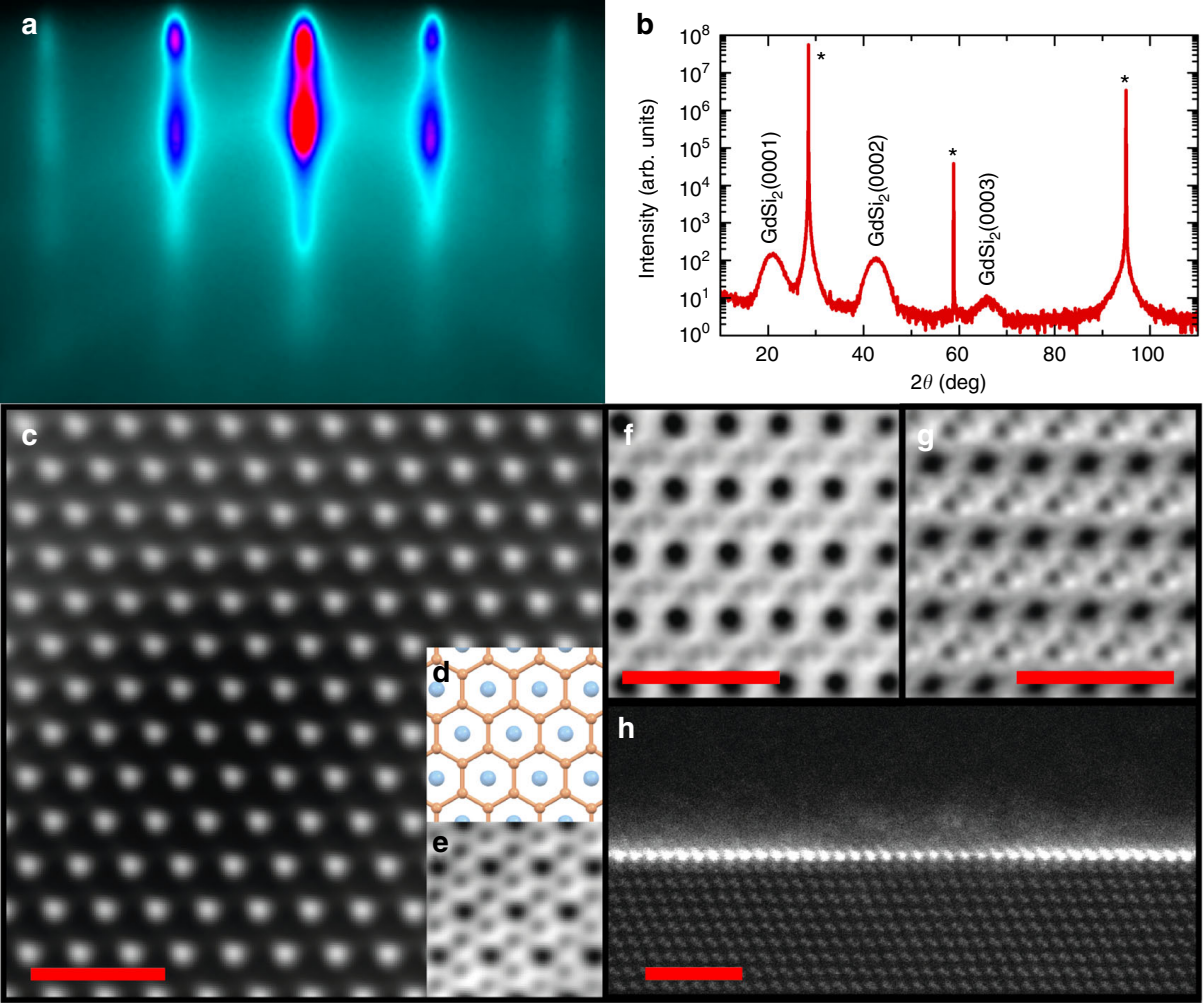
Structural characterization of silicene materials. **a** RHEED image of 4-5 ML GdSi_2_ along the $\left[ {1\bar 10} \right]$ azimuth of the Si substrate. **b** *θ*-2*θ* X-ray diffraction scan of 4-5 ML GdSi_2_ on Si(111). Asterisk denotes peaks from the Si substrate. **c** Top view of bulk GdSi_2_ (HAADF-STEM image). **d** Top view of bulk GdSi_2_ (ball-and-stick model). **e** Top view of bulk GdSi_2_ (BF-STEM image). **f** BF-STEM image of a GdSi_2_ cross-section viewed along the [110] zone axis of the Si substrate. **g** BF-STEM image of a EuSi_2_ cross-section viewed along the [110] zone axis of the Si substrate. **h** HAADF-STEM image of 1 ML of GdSi_2_ on Si(111). Scale bars, **c**, **f**, **g** 1 nm and **h** 2 nm

The structure of the films is characterized with a number of techniques. First, the state of the film surface is controlled in situ with reflection high-energy electron diffraction (RHEED). Figure 2a presents a typical RHEED image for 4-5 MLs of GdSi_2_ while more data are provided in Supplementary Fig. 1. In general, ultrathin epitaxial films of GdSi_2_ are quite flat but the surface roughness increases significantly when the film approaches its 3D limit. The structure of the grown films is analyzed ex situ with X-ray diffraction (XRD). The samples are protected by SiO_x_ and can be exposed to air. *θ*–2*θ* scans of SiO_x_/GdSi_2_/Si(111) films (see Fig. 2b and Supplementary Fig. 2) exhibit only reflexes (000*n*) from GdSi_2_ and those from the substrate, thus confirming the absence of any unwanted side products (the capping layer of SiO_x_ is amorphous and does not show up on XRD scans). The lattice parameter *c* changes significantly with thickness, from 4.1743 ± 0.0009 Å in the bulk to 4.287 ± 0.009 Å in the 2 ML film, consistent with emerging silicene buckling in ultrathin films. Then, we analyzed the peak $\left( {2\bar 202} \right)$ in a 9 ML sample employing XRD measurements for another crystallographic direction. The determined lateral lattice parameter *a* = 3.827 ± 0.017 Å, which is close to the corresponding lattice parameter of Si(111) (3.84 Å). It suggests that the silicene layers of GdSi_2_ and the Si(111) surface are lattice matched.

Further, we studied the microscopic structure of the films. Figure 2c-e presents top views on the GdSi_2_ films–high-angle annular dark-field (HAADF) and bright-field (BF) scanning transmission electron microscopy (STEM) images, as well as the matching ball-and-stick model. The honeycomb silicene structure is evident in the BF-STEM image (Fig. 2e). At the same time, cross-sectional BF-STEM images of bulk GdSi_2_ and EuSi_2_ (Fig. 2f, g, respectively) illustrate the difference in buckling of silicene sheets in the two compounds. Cross-sectional HAADF-STEM images illustrate the number of MLs in ultrathin films, as in GdSi_2_ of 1 ML in Fig. 2h and other examples (of 2, 4, and 8 MLs) provided in Supplementary Fig. 3. The values closely correspond to the growth times. The estimates based on simulation of the experimental X-ray reflectivity spectra by sets of lamellas (see Supplementary Fig. 4) are in line with the other in terms of the film thickness. However, they should be taken with a pinch of salt since the analysis is rather crude and the fit relies upon many parameters. The uncertainty in the number of MLs increases with the film thickness. The latter determines the morphology of the system. Electron microscopy does not detect any grain boundaries in the 1 ML samples. However, in thicker samples, steps at the MSi_2_/Si(111) interface show up. We attribute them to the synthesis procedure since the Si substrate serves as one of the reactants. The lateral size of the grains diminishes and approaches 15 nm for films thicker than several MLs. It does not change further, even in the bulk samples. The presence of the grains leads to non-flat surfaces, which is not uncommon for thick films on (111) substrates, reflected in changes in the RHEED patterns (Supplementary Fig. 1). Electron energy loss spectra of GdSi_2_ and EuSi_2_ confirm that the valence states of metal atoms are Gd(III) and Eu(II), respectively (Supplementary Fig. 5).

### Emerging 2D ferromagnetism

**Fig. 3:**
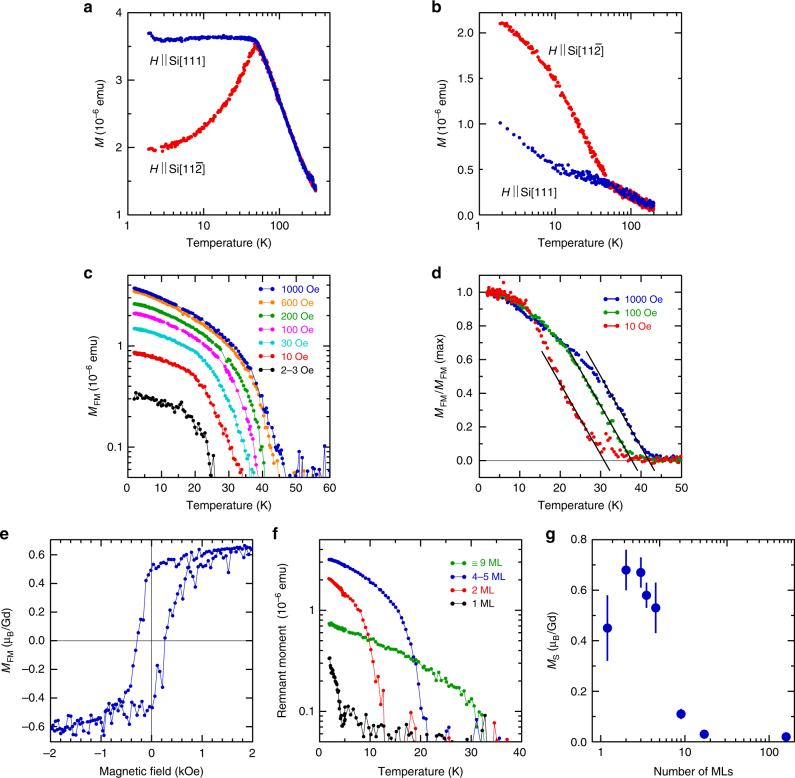
Magnetism of GdSi_2_ materials. **a** Temperature dependence of the AFM magnetic moment in bulk GdSi_2_ (≅160 ML) measured in a magnetic field 1 kOe along directions Si[111] (blue) and Si$\left[ {11\bar 2} \right]$ (red). **b** Temperature dependence of the magnetic moment in 17 ML GdSi_2_ measured in a magnetic field 1 kOe along directions Si[111] (blue) and Si$\left[ {11\bar 2} \right]$ (red). **c** Temperature dependence of the FM moment in the 4-5 ML sample of GdSi_2_ in magnetic fields 2-3 Oe (black), 10 Oe (red), 30 Oe (turquoise), 100 Oe (purple), 200 Oe (green), 600 Oe (orange), and 1 kOe (blue). **d** Temperature dependence of the normalized FM moment in the 2 ML sample of GdSi_2_ in magnetic fields 10 Oe (red), 100 Oe (green), and 1 kOe (blue). **e** Magnetic field dependence of the FM moment in the 2 ML sample of GdSi_2_ at 2 K. **f** Temperature dependence of the remnant moment (after cooling at 500 Oe) in GdSi_2_ samples of different thickness: 1 ML (black), 2 ML (red), 4-5 ML (blue), and ≅9 ML (green). **g** Dependence of the saturation moment at 2 K on the number of GdSi_2_ MLs. Error bars are s.d

The bulk sample of GdSi_2_ demonstrates magnetic behavior typical for a 3D AFM system (Fig. 3a) with the Néel temperature *T*_N_ ≈ 50 K. The curves showing the temperature dependence of the magnetic moment for external magnetic field along directions $\left[ {11\bar 2} \right]$ (red) and [111] (blue) of the Si substrate coincide above *T*_N_, as it should be in the paramagnetic state. However, below *T*_N_ the curves diverge–the behavior of the red and blue curves corresponds to an AFM magnetic moment directed along and orthogonal to the external magnetic field, respectively. That means that the out-of-plane direction is not an easy magnetization axis in GdSi_2_–magnetic moments are inclined to orient in-plane. In thinner films, the temperature dependence of magnetization changes dramatically. Indeed, the curves in the 17 ML film are quite different from those expected by scaling down from the bulk: although the transition occurs around *T*_N_ of the bulk, the temperature dependence of the magnetic moment does not correspond to the AFM state (Fig. 3b). In particular, a noticeable FM moment emerges: the temperature dependence of the in-plane moment goes up below 50 K in contrast to the AFM bulk (Fig. 3b). Further support for the FM behavior comes from measurements of the remnant magnetic moment in the 17 ML film (Supplementary Fig. 6). Further reduction of the film thickness shifts the balance further from AFM towards FM interactions. Apparently, the 17 ML structure may represent a borderline case from the bulk supporting AFM to 2D FM in ultrathin films. Such behavior bears some analogy with that of nanoscale V_5_S_8_, where magnetotransport studies suggest a breakdown of the AFM state with reduced thickness and appearance of a weak FM via an intermediate spin-glass-like state^34^. AFM-to-FM transformation is not unheard-of in magnetic systems based on metal ions with the 4*f*^7^ configuration–a well-known example is the magnetic semiconductor EuSe with transition induced by temperature shifting the balance between the nearest-neighbor and next-nearest-neighbor exchange interactions. The interplay between AFM and FM in silicene materials is predicted in a number of theoretical works: in particular, the magnetic state is controlled by strain in a Mn-silicene system^35^, by width in silicene nanoribbons^36^, by coverage of silicene with the superhalogen molecule MnCl_3_^37^. The in-plane FM signal in GdSi_2_ is an order of magnitude larger than out-of-plane signal, in contrast to Cr-based 2D magnets^11,12^. It is likely that in the 2D limit a combined effect of high magnetic anisotropy and dipolar interactions suppresses temperature fluctuations of local moments^14,15^.

As expected, the emerging ferromagnetism reveals its genuine 2D nature in low magnetic fields. Figure 3c presents the temperature dependence of the FM moment in the 4-5 ML sample of GdSi_2_ for a number of magnetic fields while Fig. 3d outlines changes in the magnetization curves in different magnetic fields in the 2 ML sample of GdSi_2_. We observe a dramatic variation of $T_{\mathrm{C}}^ \ast$ as a function of the applied field. This is a hallmark of intrinsic 2D FM: apparently the applied field strongly affects spin-wave excitation (pseudo)gap^11^, enabling long-range FM order at non-zero temperatures. The experimental increase of $T_{\mathrm{C}}^ \ast$ with the field comes naturally from $T_{\mathrm{C}}^ \ast$ being an increasing function of the spin-wave excitation (pseudo)gap^15^. It should be noticed that the fields effectively controlling the magnetic transition temperature are significantly lower than those in Cr_2_Ge_2_Te_6_^11^–a possible reason is that GdSi_2_ films correspond to the easy-plane magnetic symmetry where simple models predict a stronger sensitivity of magnetization to weak magnetic fields^15^. The magnetism is robust enough to be measured with a conventional SQUID magnetometer, in sharp contrast to the other works on intrinsic 2D FM^11,12^, employing highly sensitive magneto-optical Kerr effect microscopy. At low temperature, the *M–H* curves show a well-developed hysteresis characteristic to the FM behavior (Fig. 3e). The difference between field-cooled (FC) and zero-field-cooled (ZFC) curves (see Supplementary Fig. 7) developed at low temperature is also indicative of the magnetic order. The emerging FM strongly depends on the thickness of the GdSi_2_ film. Figure 3f provides an illustration by recruiting the example of the temperature dependence of the remnant moment. It suggests a monotonic increase of characteristic $T_{\mathrm{C}}^ \ast$ with the film thickness, similar to ref.^11^. However, the saturation magnetic moment per Gd (Fig. 3g), large in all ultrathin films, reaches its maximum at 2 ML but slightly decreases in the 1 ML films. The absolute value of the saturation moment is well short of 7 μ_B_ per Gd, expected for fully FM ordered ions with half-filled *f*-shells. The 2D character of the magnetism may contribute to the moment reduction–ref.^11^ reports stronger than linear decrease of the effective Kerr signal with diminishing thickness in a layered ferromagnet. Among other possible reasons for the reduced moment are the non-ionic character of the silicides and likely AFM fluctuations arising from competing magnetic interactions.

### Transport properties

**Fig. 4:**
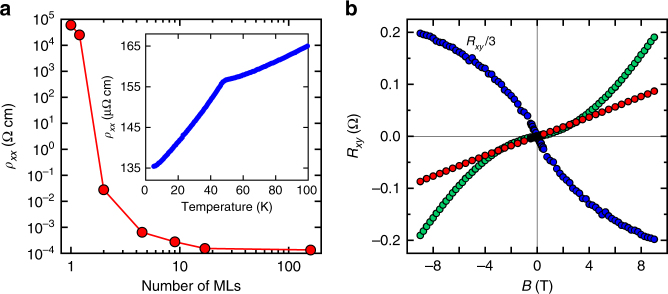
Transport properties of GdSi_2_ materials. **a** Dependence of longitudinal resistivity at 2 K on the number of GdSi_2_ MLs. Inset: Temperature dependence of resistivity in the bulk GdSi_2_ sample (≅160 ML) showing an AFM transition around 50 K. **b** Hall resistance at 2 K for GdSi_2_ samples of different thickness: ≅160 ML (red), ≅17 ML (green), and ≅9 ML (blue). The samples with ≅17 ML and ≅9 ML thickness demonstrate a pronounced anomalous Hall effect–strongly non-linear dependence *R*_xy_(*B*)

The profound changes in the magnetic state with the film thickness must leave an imprint in transport properties of the films. Figure 4a presents longitudinal resistivity *ρ*_*xx*_ measured at 2 K in structures with different numbers of GdSi_2_ MLs. The result is quite remarkable: *ρ*_*xx*_ is almost 9 orders of magnitude larger in 1 ML than in the bulk (≅160 ML). This fact shows that the behavior of electrons confined in an ultrathin layer is utterly different from that in the bulk. Both dimensionality effect and strong electron correlations lead to dramatic changes of the electronic structure. The downside is that this system presents an enormous challenge to any sort of modeling or electronic structure computations based on the one-electron approach. Electron transport measurements confirm the thickness-dependent nature of the magnetism in GdSi_2_. The temperature dependence *ρ*_*xx*_(*T*) exhibits a prominent feature at *T*_N_ in bulk GdSi_2_ (inset of Fig. 4a)–below *T*_N_ a spin gap opens, magnetic fluctuations are suppressed and the resistivity changes slope towards a stronger decrease. However, this feature of the AFM state is progressively suppressed as soon as the film thickness is reduced (Supplementary Fig. 8).

The anomalous Hall effect (AHE) is suggested as a useful instrument to probe magnetic ordering in 2D crystals^34^. It is observed in materials with broken time-reversal symmetry such as FM conductors and relies on spin-orbit coupling^38^. The experimental signature of the AHE is a sharp increase of *R*_*xy*_(*B*) at low field *B*, which crosses over into a linear region under high magnetic field. This initial increase of *R*_*xy*_(*B*) is due to the saturation of magnetization of the sample under external field. Transport measurements at 2 K reveal that Hall resistance is linear in bulk GdSi_2_, without any signs of the AHE. However, a pronounced AHE appears in thin samples (see Fig. 4b), thus indicating the emergence of FM moments. The change of the behavior of Hall curves (Fig. 4b) from hole-like in the bulk and 17 ML samples to electron-like in the 9 ML sample is a further indication of significant transformation in the electronic structure with film thickness. It is quite possible that more than one type of carriers is present (as common in silicides), which is consistent with the observed non-parabolic magnetoresistance. It is important that the slope of the curves is not related to the AHE–the same transformation occurs at 50 K where *R*_*xy*_*(B)* is linear for all samples.

### Control experiments

The experiments with GdSi_2_ leave some questions unanswered. First, the role of defects in the transformation from 3D AFM to 2D FM needs to be clarified. Free-standing silicene is known to be buckled^17^. However, in the silicide form planar Si layers are much more common–they are formed by many lanthanides and actinides. Bulk rare-earth silicides with planar Si layers are known to relax the ensuing strains with the help of a significant amount of vacancies^39^ which have a tendency to order. Indeed, Rutherford backscattering study of GdSi_2_ (Supplementary Fig. 9) confirms that our bulk films contain Si vacancies. However, the corresponding ultrathin films with buckled silicene layers are known to be defect-free^39^. On the other hand, the relevance of the silicene structure to the 2D FM order is not yet demonstrated. Thus, a number of control experiments are required to establish the general and intrinsic character of the phenomenon and elucidate the role of the silicene lattice.

**Fig. 5:**
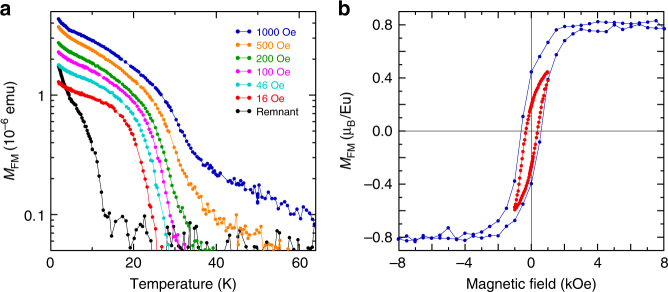
2D magnetism of EuSi_2_ materials. **a** Temperature dependence of the FM moment in the 4 ML sample of EuSi_2_ in magnetic fields 16 Oe (red), 46 Oe (turquoise), 100 Oe (purple), 200 Oe (green), 500 Oe (orange) and 1 kOe (blue), as well as remnant moment (black). **b** Magnetic field dependence of the FM moment in the 4 ML sample of EuSi_2_ at 2 K

Silicene-based polymorph of EuSi_2_^28^ is structurally similar to GdSi_2_ but boasts buckled silicene layers irrespective of thickness, similar to CaSi_2_^25^ and SrSi_2_^27^. Therefore, the problems with defects and change of the buckling are absent in EuSi_2_. Similar to GdSi_2_, the study of magnetism in EuSi_2_ films of different thickness reveals transformation of the 3D AFM order into 2D FM in ultrathin films. Ferromagnetism in the 4 ML sample of EuSi_2_ is illustrated employing temperature dependence of the FM moment in low magnetic fields (Fig. 5a), magnetic hysteresis loops (Fig. 5b), and the divergence of FC and ZFC curves (Supplementary Fig. 7). The strong analogy with GdSi_2_ is evident; even the saturation magnetic moments are close. Transport measurements show the presence of AHE in ultrathin films of EuSi_2_; the effect becomes larger for decreased thickness (Supplementary Fig. 10). Thus, the phenomenon of intrinsic 2D FM in silicene materials is quite general and not limited to one particular compound.

It is important to establish that impurities are not responsible for the reported effects. A general argument is that impurities can hardly explain the observed qualitative features, such as thickness-dependent AFM-to-FM transition, emergence of 2D ferromagnetism, the AHE in transport measurements, strong dependence of $T_{\mathrm{C}}^ \ast$ on weak magnetic fields, as well as the large value of the observed saturation moment and the similarity between Gd- and Eu-based silicene materials (chemically similar compounds of Gd and Eu exhibit different magnetic properties). Impurities do not show up in the XRD scans and electron microscopy images for all MSi_2_ films. More specifically, one can suggest free metals and oxides as potential impurities. However, free metal impurities can hardly survive at high temperature of the synthesis in a close vicinity of virtually unlimited source of silicon. Also, formation of metal impurities is less probable in the case of few ML films (where the FM behavior emerges) than in the bulk. Formation of metal oxides requires an oxygen reactant; the only oxygen-containing compound in the system is the SiO_x_ capping. SiO_x_ is deposited at room temperature when metal is exhausted in the silicide formation, which precludes formation of oxides. Nevertheless, we carried out a control experiment by synthesizing a heterostructure of 3 ML of EuSi_2_ on Si(111), capped with 200 Å of Al. This structure prepared in ultrahigh vacuum (UHV) is oxygen-free but demonstrates very similar XRD scans and magnetic properties as an equivalent film capped with SiO_x_ (Supplementary Fig. 11).

**Fig. 6:**
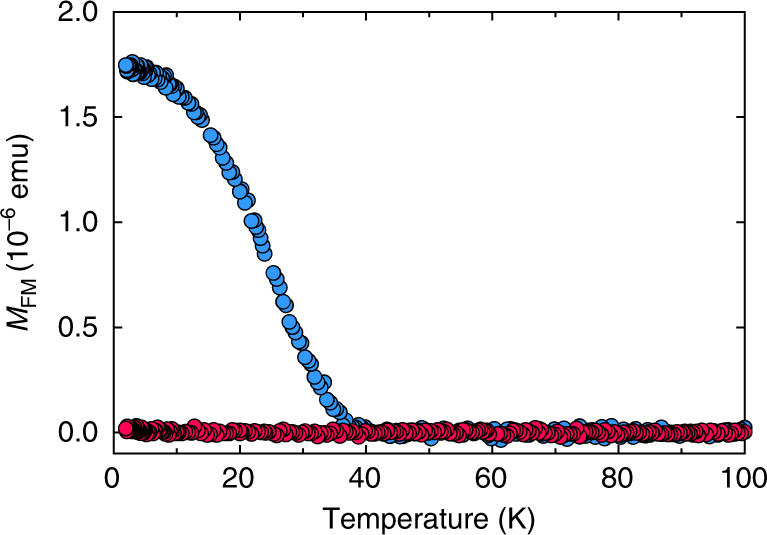
2D magnetism of GdSi_2_ polymorphs. Temperature dependence of magnetic moment in a magnetic field of 50 Oe: tetragonal GdSi_2_ of 2 unit cell thickness (red) as compared with 2 ML silicene-based GdSi_2_ (blue)

To prove the importance of silicene in 2D magnetism we synthesized another polymorph of GdSi_2_, tetragonal, stabilized by the Si(001) substrate. In contrast to the silicene-based polymorph, ultrathin films of tetragonal GdSi_2_ show no magnetism within the experimental error (see Fig. 6 for comparison of the two polymorphs). The epitaxial stabilization by the substrate influences the magnetism strongly but indirectly via drastic changes in the atomic arrangement of the MSi_2_ structure. To rule out a direct influence of the substrate on the observed FM behavior we have grown a film of 4 MLs of EuSi_2_ on the SrSi_2_/Si(111) template–the films SiO_x_/EuSi_2_/Si(111) and SiO_x_/EuSi_2_/SrSi_2_/Si(111) (of the same thickness of the EuSi_2_ layer) exhibit very similar FM signals. Therefore, silicene lattice is instrumental in the observed 2D FM order. We do not suggest silicene to be the seat of magnetism. Ultrathin films of layered SrSi_2_ (isomorphous to EuSi_2_) with thickness 4 ML and 8 ML do not show any FM behavior (see Supplementary Fig. 12 for comparison of SrSi_2_ and EuSi_2_). However, the extended π-system of silicene can mediate long-range magnetic interactions because the *p*-states are much more delocalized than *f*-electrons. Otherwise, it is possible that silicene supports magnetism simply by favouring a specific spatial configuration of M atoms (local moments). As explained above, chemically functionalized silicene is suggested to promote competition between FM and AFM phases^35–37^. It is likely that M atoms interact strongly with π-states of silicene, σ-orbitals are less affected and may dominate the electronic states near the Fermi level. Chemical functionalization naturally changes the topological properties of the system. In particular, *s*-*p* band inversion can bring non-trivial topological states in heavy 2D-Xenes^17^; one-side-saturated 2D-Xenes are predicted to become quantum anomalous Hall insulators^40^.

## Discussion

Silicene holds high promise today, a promise of new physical phenomena and new device concepts for nanoelectronics and particularly spintronics. In reality, however, silicene turns into a fertile playground for theoretical research with experimental studies virtually limited to synthesis and structural and spectroscopic characterization of supported 2D Si structures. The quest to actually demonstrate any useful properties of silicene is now on the rise. Our approach is to start from bulk silicene materials, i.e. layered compounds formed by functionalized silicene lattices. The study of magnetism in silicene-based EuSi_2_ and GdSi_2_ shows the common dimensionality effect, which is absent in the non-silicene polymorph. It manifests the active role of the silicene lattice. Robust intrinsic 2D FM emerging from AFM of the bulk reveals competition between fundamental magnetic interactions. In ultrathin films, low magnetic fields provide an effective control over spin-wave excitation spectrum and, thus, the transition temperature $T_{\mathrm{C}}^ \ast$. It highlights the fundamental difference between the results of the present work and those of ref.^28^ which reports the first synthesis of the 3D parent compound EuSi_2_. The magnetic state of EuSi_2_ films approaching bulk is dominated by antiferromagnetism^28^. Only controlled synthesis of films formed by a few MLs of the silicide reveals the thickness-dependent AFM-to-FM transformation, 2D nature of the emerging ferromagnetism, the role of the silicene-based structure. The new results for EuSi_2_ combined with those for GdSi_2_ (not studied in ref.^28^) suggest the discovery of a class of 2D FM silicene materials, especially taking into account that a number of rare-earth materials are isostructural to GdSi_2_–in particular, MSi_2_ with M from Gd till Tm^39^. Further work is necessary to synthesize and determine magnetic properties of those other silicene compounds. The rare-earth magnets MSi_2_ cover the same temperature range as Cr-based 2D FM materials^11,12^ but differ in other important ways such as the in-plane character of their magnetism and its robustness allowing for studies with a conventional magnetometer. Silicene termination of MSi_2_ films makes them good candidates for integration with van der Waals materials leading to novel types of heterostructures. Direct incorporation of the silicene materials with Si is particularly enticing for spintronic applications. We envisage that our results would provide a platform for studies of 2D FM in layered structures leading to new discoveries and applications in this exciting field of research.

## Methods

### Synthesis

The samples of MSi_2_ materials are grown in the Riber Compact 12 system for molecular beam epitaxy furnished with a UHV system comprising cryopump Cryo-Torr 8 (Brooks CTI Cryogenics), Gamma Vacuum TiTan Ion Pump, a titanium sublimation pump and cryopanels cooled by liquid N_2_. Base pressure of less than 10^−10^ Torr is maintained in the growth chamber. Silicene-based GdSi_2_ and EuSi_2_ are grown on Si(111) while tetragonal GdSi_2_ is grown on Si(001). Both types of substrates are high-Ohmic with miscut angles not exceeding 0.5°. All wafers are 1 inch × 1 inch squares. 4N Eu, 4N Gd and SiO_x_ (capping material) are supplied from Knudsen cell effusion sources. The intensity of molecular beams is monitored with Bayard–Alpert ionization gauge fitted at the substrate site. Temperature of the substrate is controlled with PhotriX ML-AAPX/090 infrared pyrometer (LumaSense Technologies) operating at the 0.9 μm wavelength. During the growth, the surface of the film is analyzed in situ with RHEED fitted with kSA 400 Analytical RHEED System (k-Space Associates, Inc.).

Synthesis starts with removal of the natural surface oxide by heating the Si substrate at 950 °C. Then, the basic chemical reaction

1 $$M + 2\mathrm{Si} = M\mathrm{Si}_2$$

is carried out by directing a flow of metal atoms (*M* = Gd or Eu) at the Si substrate. Tetragonal GdSi_2_ is synthesized by heating the effusion cell to 1210 °C and the Si(001) substrate to 420 °C. It corresponds to a 1·10^−8^ Torr flow of Gd and a growth rate of 7 Å/min. Layered GdSi_2_ is grown at the same flow of Gd but the Si(111) substrate is held at 400 °C, which corresponds to a growth rate of 1 ML at 37 s. It should be noted that for bulk samples, after 7 min of the growth, the temperature of the substrate is raised to 550 °C. This two-step procedure is used to improve the quality of the bulk samples: low temperature leads to polycrystalline films while high temperature causes some porosity of the film. EuSi_2_ is grown using a different protocol. First, Eu is deposited at a cleaned Si(111) substrate at room temperature. The deposition rate is 9 Å/min, which corresponds to an effusion cell temperature of 410 °C (*P*_Eu_ = 5·10^−8^ Torr). Then, the film is heated to 340 °C and annealed for 5 min. This technique allows for growth of layered EuSi_2_ directly on Si, in contrast to high-temperature growth in ref.^28^ requiring a template of layered SrSi_2_. Finally, to avoid degradation by air the reactive surfaces of the MSi_2_ films are protected by a capping layer of SiO_x_. This layer is deposited after cooling the MSi_2_/Si film to room temperature in the growth chamber held under UHV. An effusion cell with SiO_x_ is heated to 950 °C which results in a SiO_x_ flow of 2·10^−8^ Torr. We grow 200 Å of the SiO_x_ capping which is more than enough for protection of the film.

### Structural characterization

The structure of all the films is determined with a number of techniques. The characterization already starts in the growth chamber, where RHEED images give a faithful representation of the state of the film surface. X-ray diffraction and reflectivity studies of the MSi_2_ samples are carried out using Rigaku Smartlab 9 kW diffractometer (CuK_α1_ X-ray source with the wavelength 1.54056 Å). All spectra are recorded in the high-resolution mode using a double-bounce monochromator Ge (220) (+−), a collimating parabolic mirror, and a system of collimating slits. The overall resolution Δ*q*_*z*_ is 0.0004 Å^−1^. All the optical scheme parameters–the beam divergence after the optical system, spectral distribution of the incident radiation, and dispersion–are used in the analysis of the experimental data. All (000*n*) peaks are taken into account to estimate the lattice parameters. XRR curves are fitted on the basis of Abeles matrix method using the reduced *χ*^2^ statistics.

Another technique of structural characterization is electron microscopy. Cross-sectional samples for analytical TEM/STEM are prepared in a Helios NanoLab 600i (FEI) Scanning Electron Microscope (SEM)/Focus Ion Beam (FIB) dual beam system equipped with an Omniprobe micromanipulator and a gas injector for Pt deposition. First, the surface of the sample is covered with a 2 μm layer of Pt. FIB milling with 30 keV Ga^+^ ions results in 2 μm cross-sections of approximately 5 × 5 μm^2^ area. For electron transparency, the samples are thinned with 5 keV Ga^+^ ions. Final cleaning is achieved with a 2 keV Ga^+^ ion beam. Alternatively, to get the top view, samples are mechanically thinned from the substrate side to a 50 μm thickness; then the substrate is further removed by Ar^+^ ion beams in PIPS system (Gatan). The atomic structure of silicides is studied with TEM/STEM Titan 80-300 microscope (FEI) with a 0.8 Å STEM probe size and an EELS energy resolution of 0.75 eV, equipped with HAADF and BF detectors, a spherical aberration (Cs) corrector, a Si(Li) energy dispersive X-ray spectrometer (Phoenix System, EDAX), and a post-column Gatan energy filter (GIF). The images are analyzed with the Digital Micrograph (Gatan) and Tecnai Imaging and Analysis (FEI) software.

Rutherford backscattering experiments are carried out at the accelerator 3MV Tandetron 4130 MC +(HC) using ^4^He^2+^ ions with an energy of 2 MeV. The measurements employ the standard IBM scattering configuration, where the incident beam, surface normal and detected beam are all coplanar. The angle between the incident beam and surface normal is 40°; the detector is 10° closer to the surface normal. The sample is tilted to increase the effective thickness of the film.

### Magnetism and electron transport

Magnetic properties of the films are determined with MPMS XL-7 Superconducting Quantum Interference Device (Quantum Design). The samples are mounted in plastic straws orienting the surface of the film with respect to the applied magnetic field with accuracy better than 2°. The measurements are carried out using the reciprocating sample option (RSO).

The measured films are extremely thin. At this featherweight level, the contribution to the signal from the Si substrate cannot be neglected. We use two approaches to extract the FM moment *M*_FM_. One approach is to subtract the diamagnetic moment of the Si substrate, measured in a separate experiment; then, the low-temperature moment is shifted by subtracting its value at 100 K. An alternative is to remove all contributions linear in magnetic field. We take measurements at some field H′ high enough that the FM contribution is saturated and small but the other contributions are far from saturation. Then, the FM moment is estimated as

2 $$M_{\mathrm{FM}}\left( H \right) = M\left( H \right) - M\left( {H\prime } \right)\frac{H}{{H\prime }} \cdot$$

The two schemes provide similar results. It proves the self-consistency and correctness of the subtraction. It is important that the presented remnant moments and difference between FC and ZFC curves do not depend on the subtraction. The FM moments are observed already in tiny magnetic fields where the subtracted contribution is very small. Also, the substrate susceptibility in the region of *T* between 20 K and 50 K (embracing all $T_{\mathrm{C}}^ \ast$) is constant which means that the subtraction does not affect the shape of the *M(T)* curves.

Transport measurements of the resistivity and Hall effect are carried out in the van der Pauw configuration using Lake Shore 9709 A Hall effect measurement system. In all cases, square samples are cut to a lateral dimension around 5 mm. Ohmic contacts to the films are made by placing an Ag–Sn–Ga alloy on each terminal.

### Data availability

The data that support the findings of this study are available from the corresponding author upon reasonable request.

## Electronic supplementary material

Supplementary Information
