# Supplementary material for: Emerging two-dimensional ferromagnetism in silicene materials
Source: Nat Commun. 2018 Apr 26;9:1672. doi: 10.1038/s41467-018-04012-2 (PMC5920055; doi:10.1038/s41467-018-04012-2)
Supplement: Supplementary file 1 — Supplementary Information [file 41467_2018_4012_MOESM1_ESM.pdf]

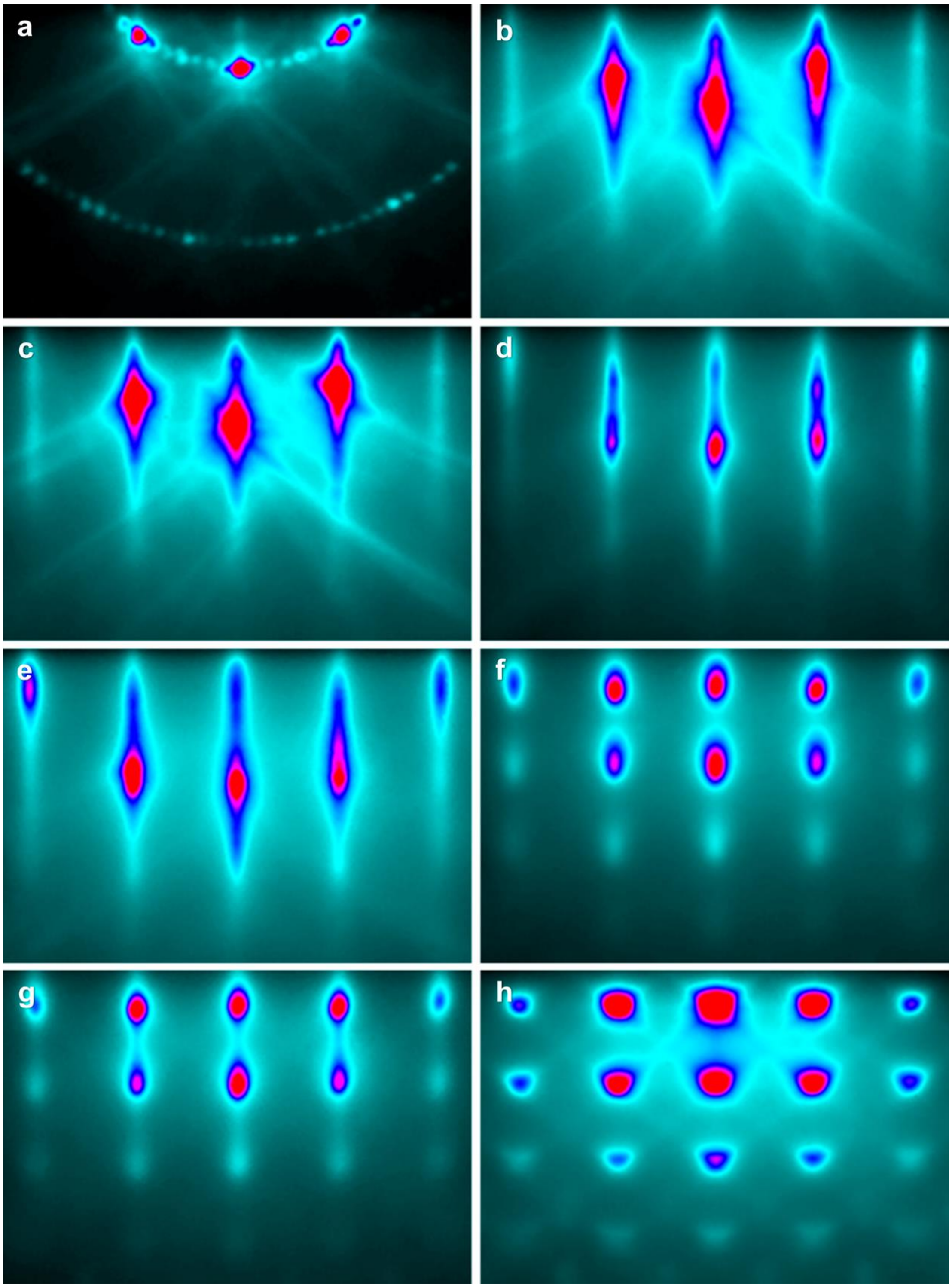

Supplementary Figure 1. RHEED images of Si(111) and $GdSi_2$. (**a**) Reconstructed Si(111) surface; (**b-h**) $GdSi_2$ with thickness 1 ML (**b**), 1-2 ML (**c**), 2 ML (**d**), 3 ML (**e**), ≅ 9 ML (**f**), ≅ 17 ML (**g**), ≅ 160 ML (**h**). All images are along the $[1\bar{1}0]$ azimuth of the Si substrate.

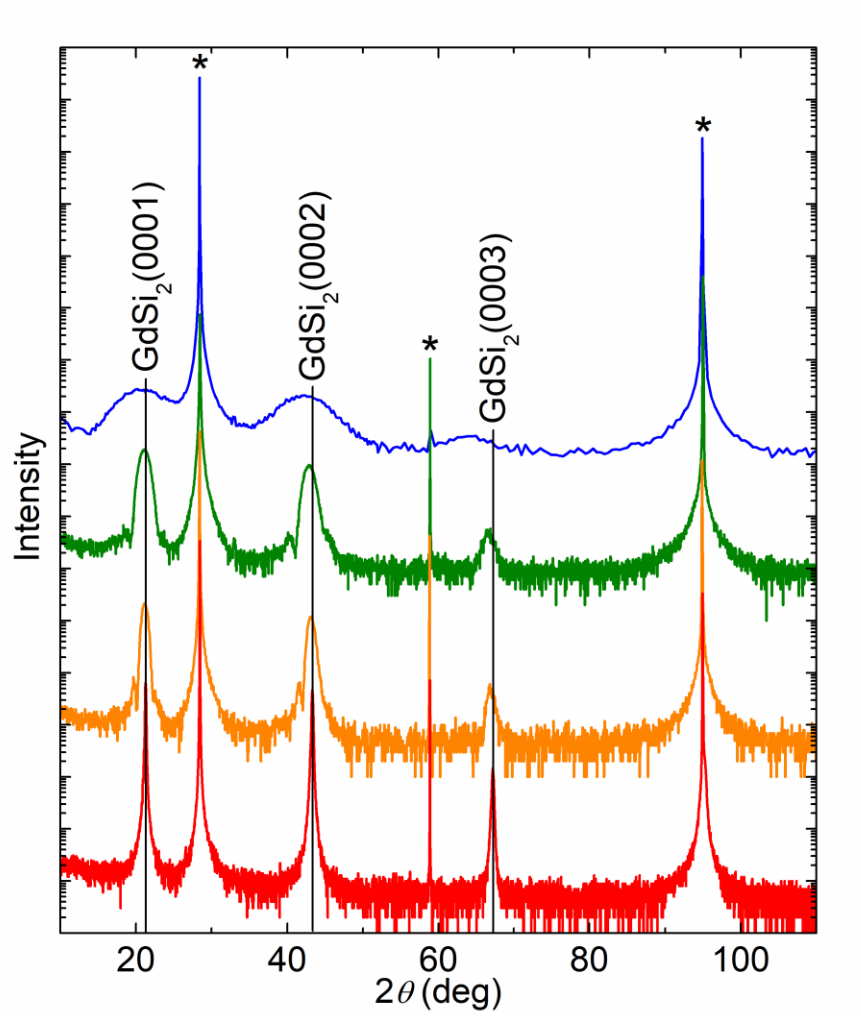

Supplementary Figure 2. X-ray diffraction study of $GdSi_2$ on Si(111). θ–2θ diffraction scans for films with thickness 2 ML (blue), ≅ 9 ML (green), ≅ 17 ML (orange), bulk (red). Asterisk denotes peaks from the Si substrate.

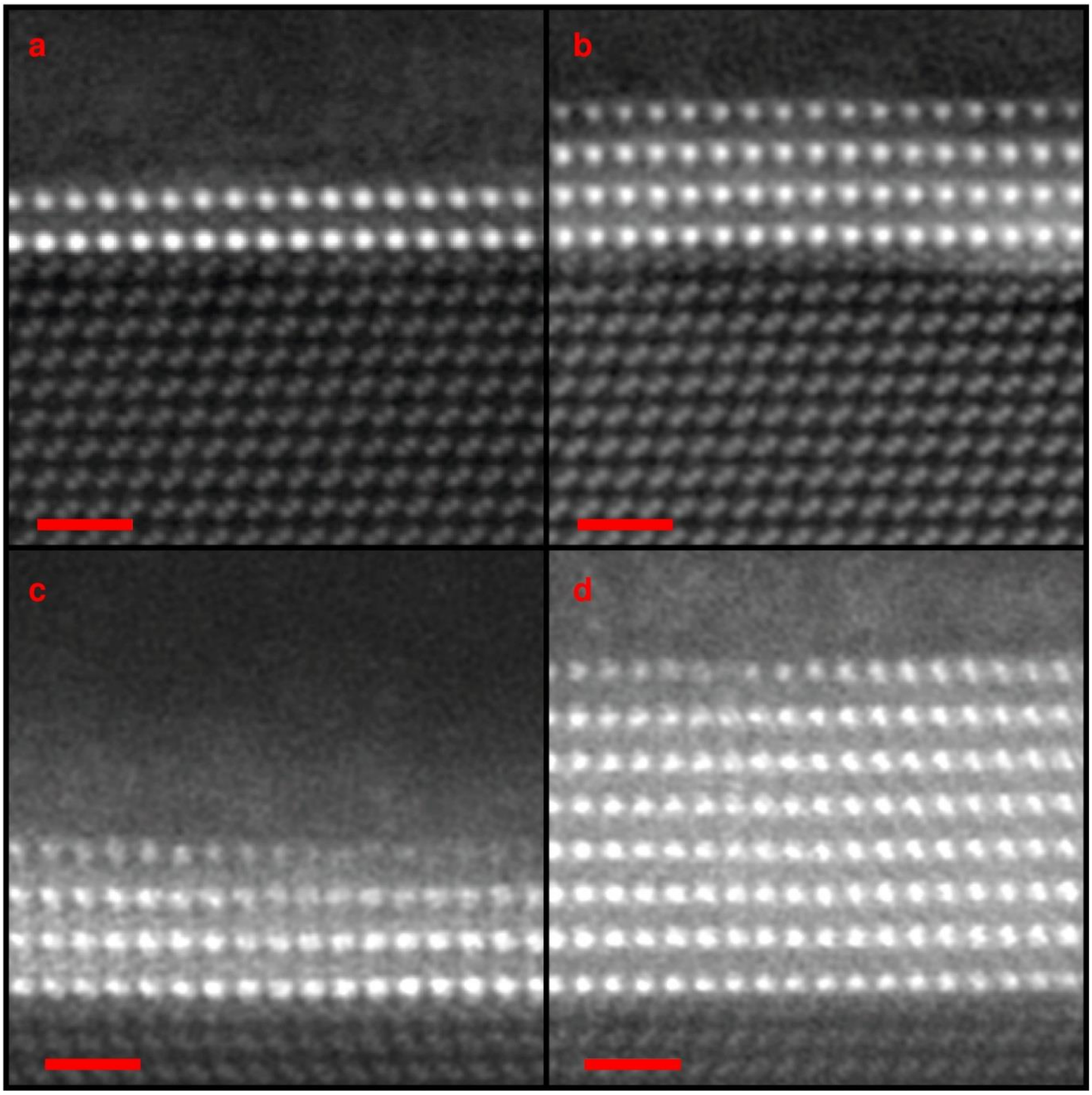

Supplementary Figure 3. Electron microscopy of ultrathin films. Cross-sectional HAADF-STEM images (viewed along the [110] zone axis of the Si substrate) of $GdSi_2$ films of (**a**) 2 ML and (**b**) 4-5 ML thickness and $EuSi_2$ films of (**c**) 4 ML and (**d**) ≅8 ML thickness. Scale bar, 1 nm.

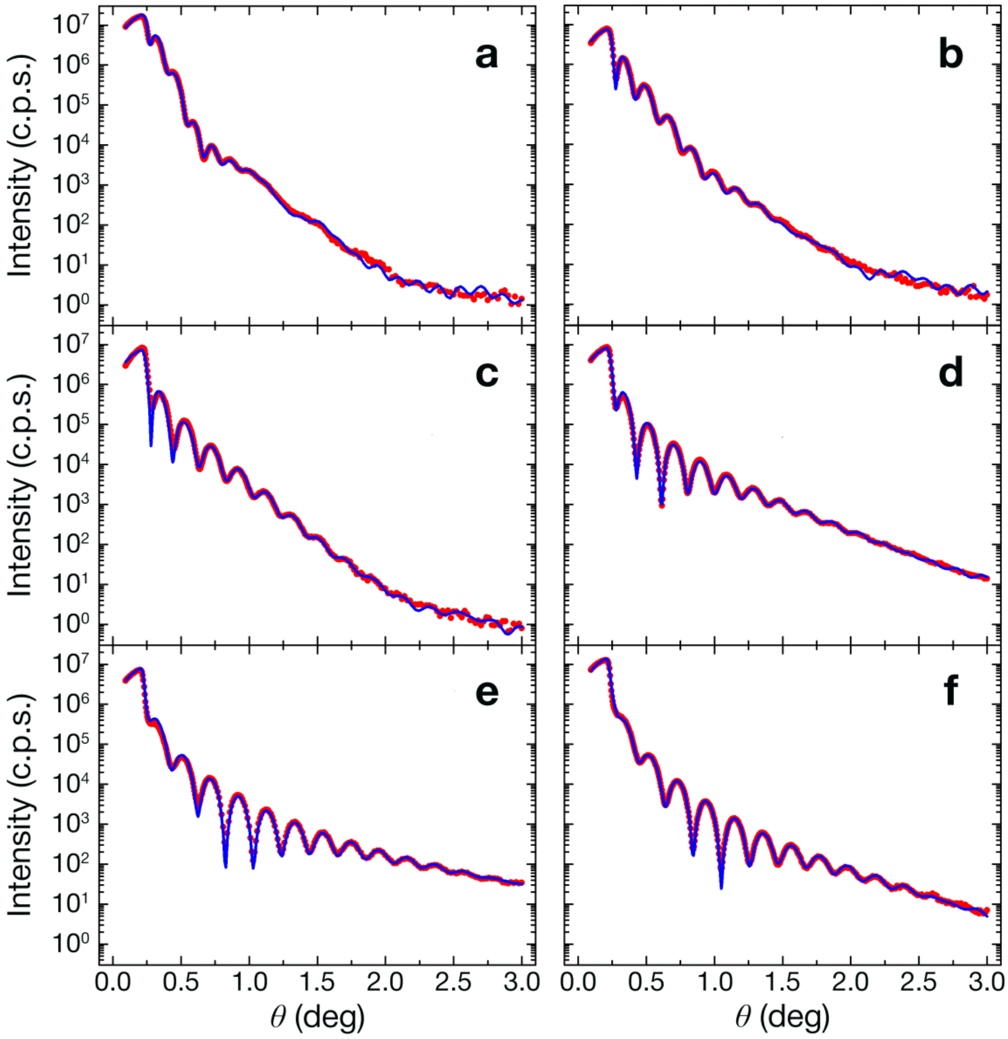

Supplementary Figure 4. X-ray reflectivity study of the films. Experimental curves (red) and theoretical fits (blue) for $GdSi_2$ on Si(111): (**a**) ≅ 17 ML; (**b**) ≅ 9 ML; (**c**) 4-5 ML; (**d**) 2 ML; (**e**) 1-2 ML; (**f**) 1 ML. The theoretical analysis relies upon a model based on lamellas with fitted parameters. This model is rather crude and the applicability of the analysis is limited.

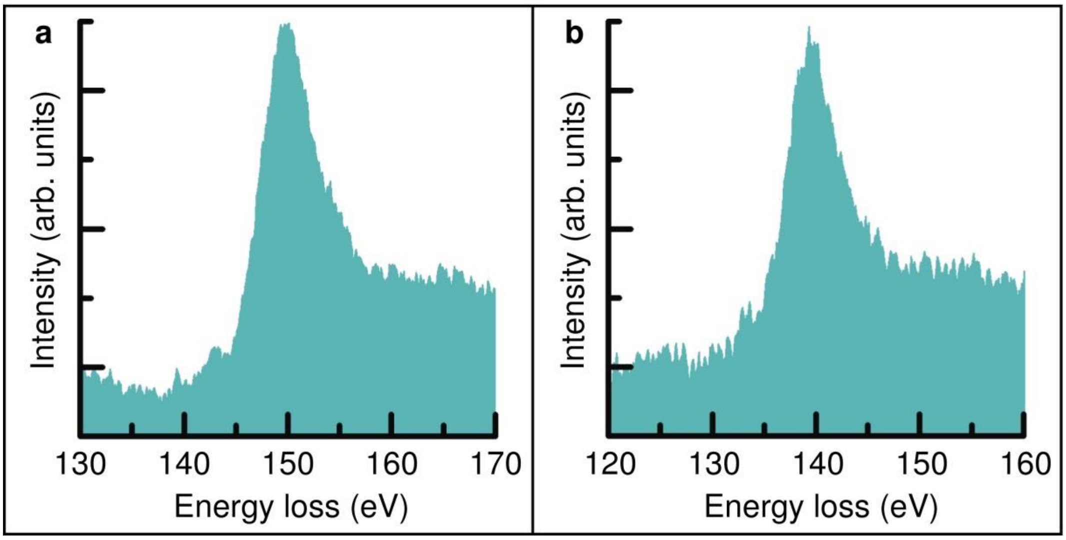

Supplementary Figure 5. Electron energy loss spectra of the films. The spectra of (**a**) ≅9 ML of $GdSi_2$ and (**b**) ≅8 ML of $EuSi_2$ show that the valence states of metal atoms are Gd(III) and Eu(II), respectively. Background is subtracted.

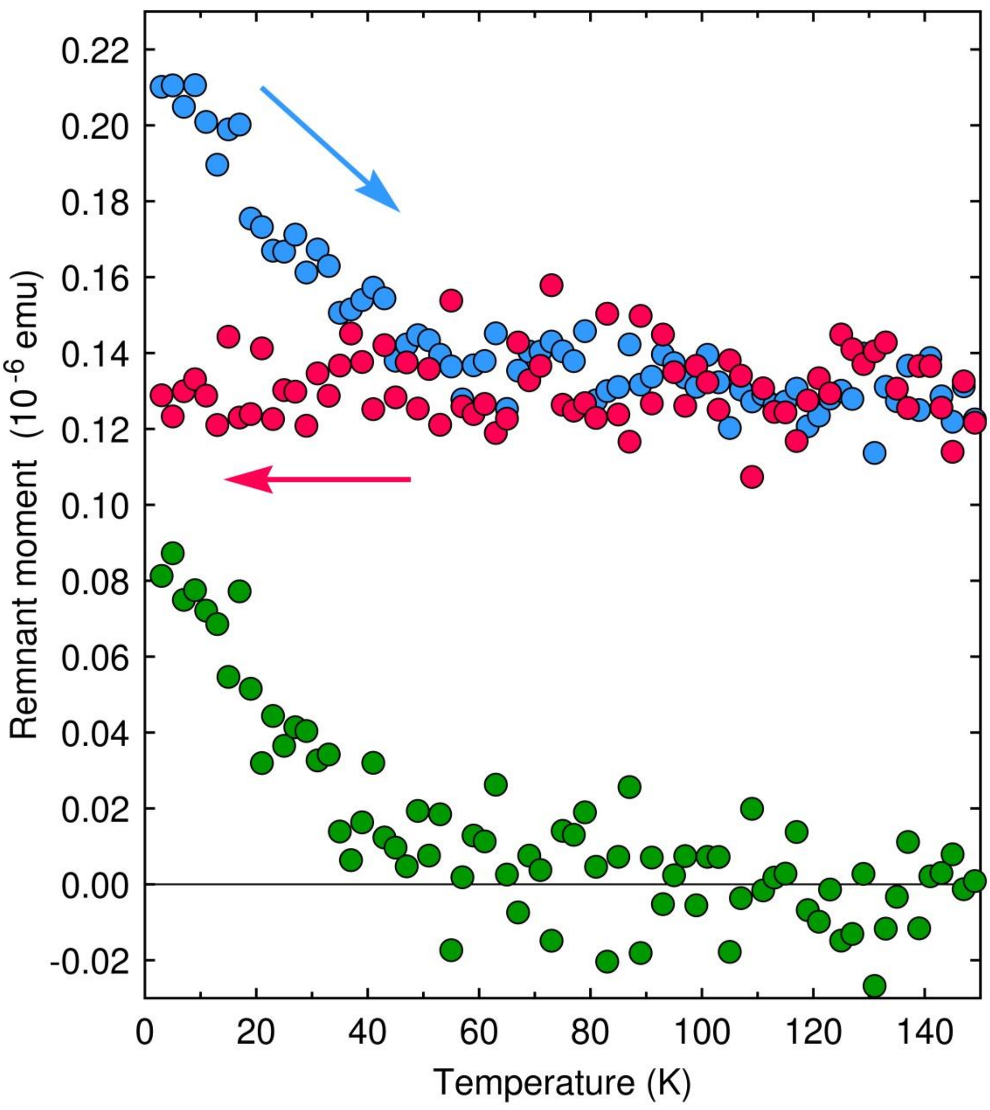

Supplementary Figure 6. Temperature dependence of the remnant moment. The moment is measured (after cooling in a magnetic field of 500 Oe) for the 17 ML sample of $GdSi_2$: heating up (blue), subsequent cooling down (red), the difference between them (green). Compare with Fig. 3f of the main text.

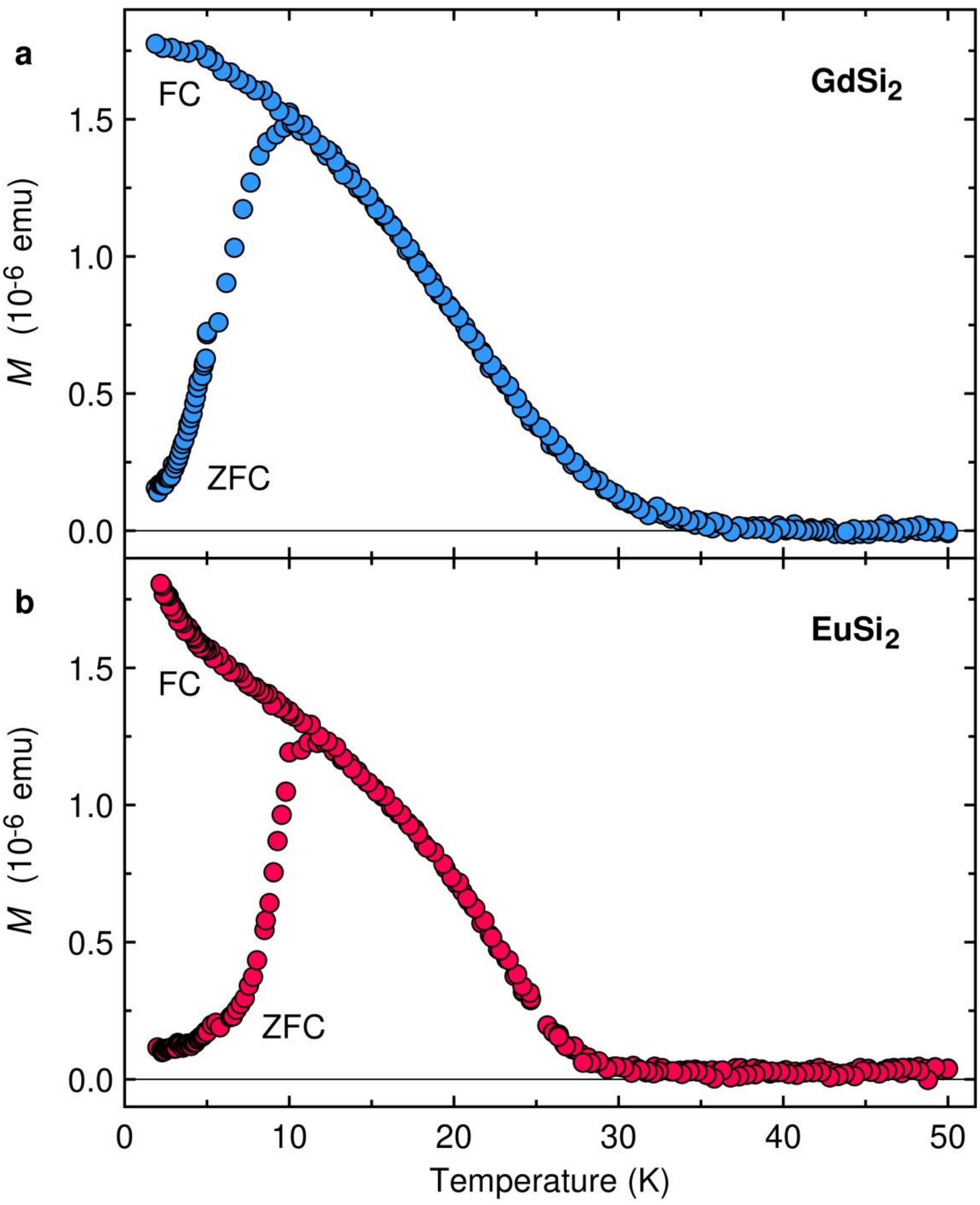

Supplementary Figure 7. Magnetic measurements of ultrathin films. Zero-field-cooled (ZFC) and field-cooled (FC) curves (50 Oe) for (**a**) 2 ML $GdSi_2$; (**b**) 1-2 ML $EuSi_2$.

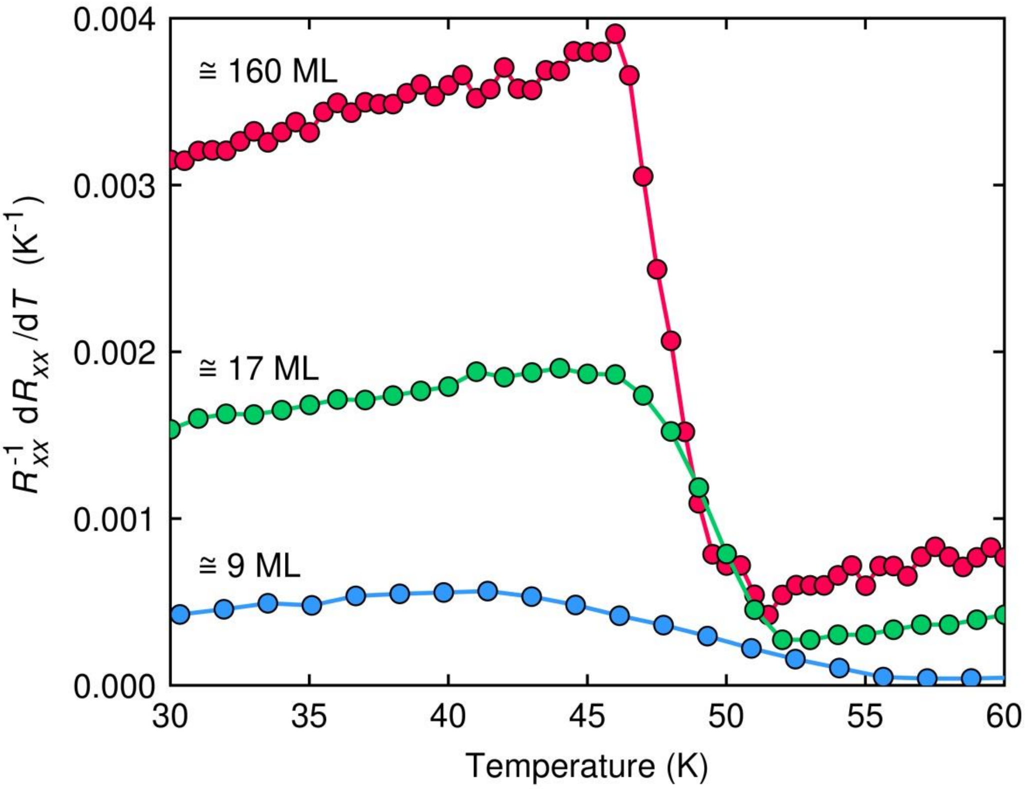

Supplementary Figure 8. Temperature dependence of resistance. Temperature coefficient of resistance for $GdSi_2$ films with a thickness ≅ 160 ML (red); ≅ 17 ML (green) and ≅ 9 ML (blue).

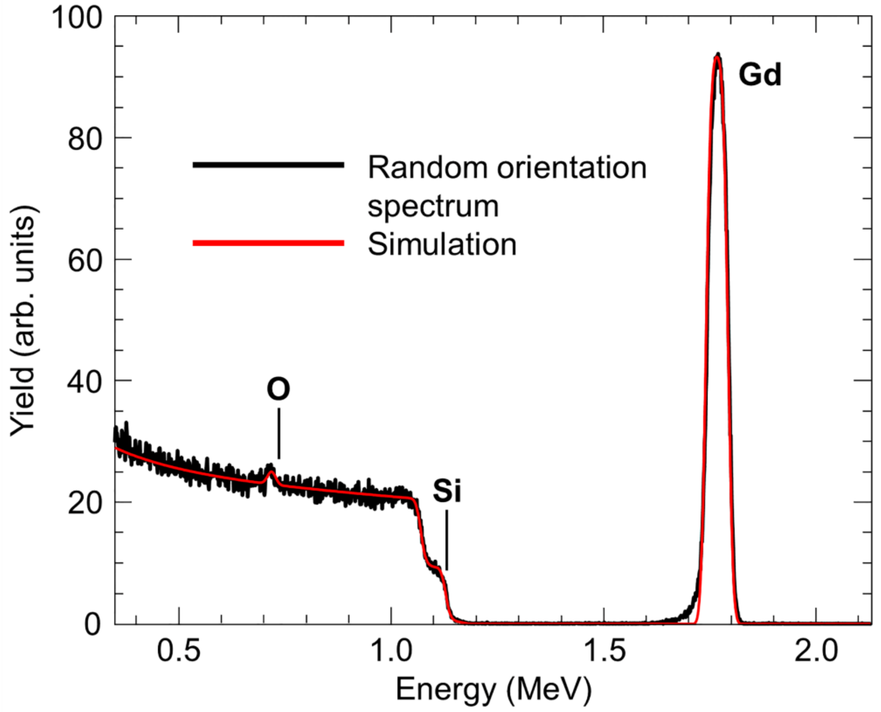

Supplementary Figure 9. Rutherford backscattering spectrum. The spectrum in random orientation (black) and its simulation (red) for bulk $GdSi_2$ on Si(111) reveal the $GdSi_{1.84}$ stoichiometry.

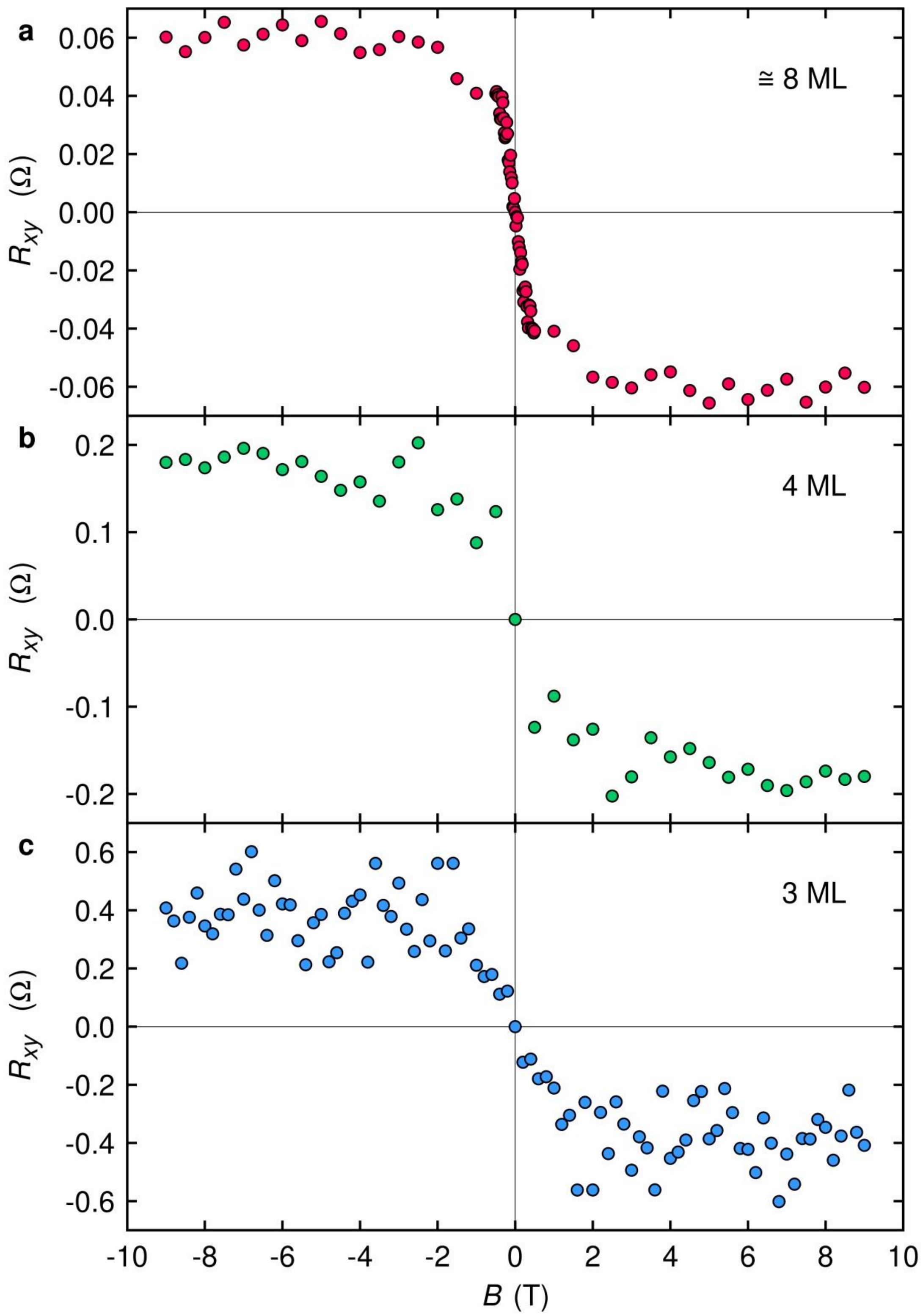

Supplementary Figure 10. Anomalous Hall effect. Hall resistance at 2 K demonstrates anomalous Hall effect in $EuSi_2$ on Si(111): (**a**) ≅8 ML (red); (**b**) 4 ML (green) and (**c**) 3 ML (blue). Note different scale of the effect for different film thickness.

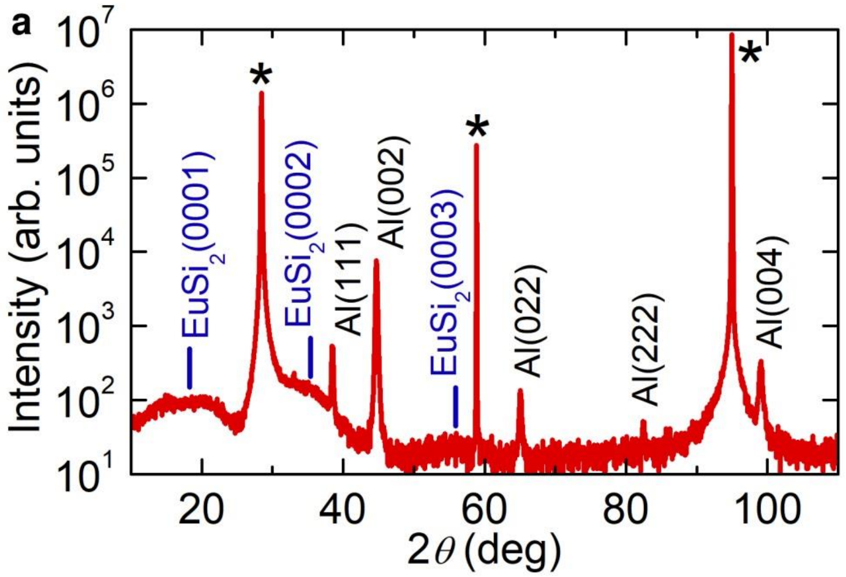

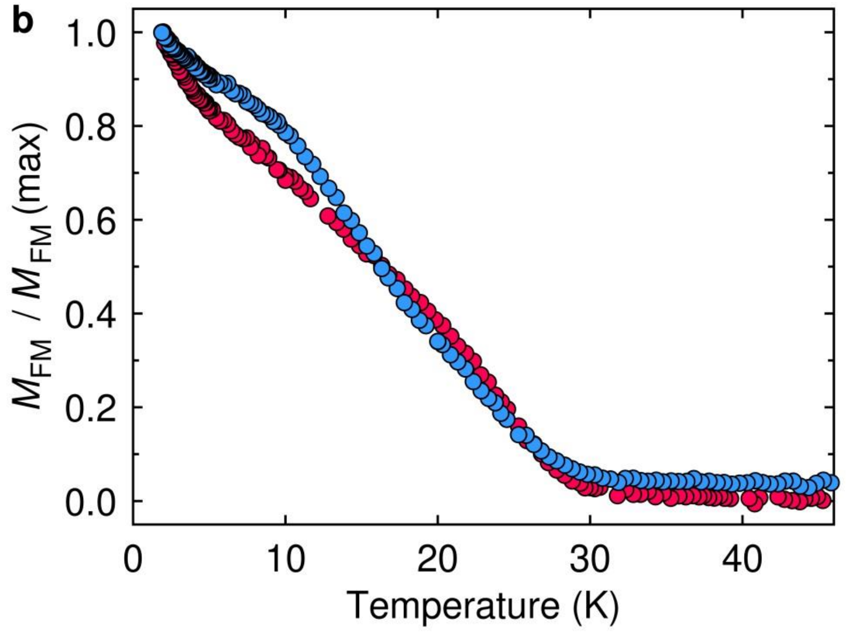

Supplementary Figure 11. Study of the film of 3 ML of $EuSi_2$ on Si(111) capped with 20 nm of Al. (**a**) θ–2θ X-ray diffraction scan showing peaks from $EuSi_2$ (the peaks are rather broad due to a very small thickness of the film), polycrystalline Al and the Si substrate (denoted by asterisk). (**b**) Comparison of 3 ML $EuSi_2$ films with different capping: temperature dependence of magnetic moment in a magnetic field of 100 Oe: SiO_x/$EuSi_2$/Si(111) (red) and Al/$EuSi_2$/Si(111) (blue). The effective Curie temperatures are quite close; the difference in the form of the curves probably comes from a slight variation of growth parameters and resulting film thicknesses in two separate syntheses.

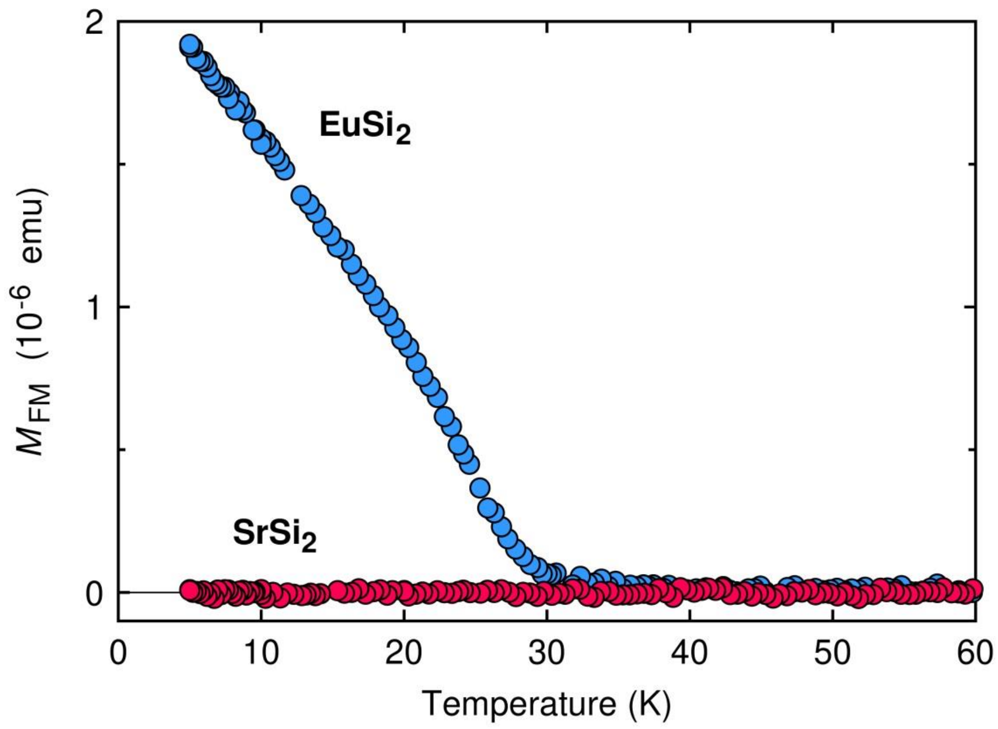

Supplementary Figure 12. Comparison of magnetism in silicene-based silicides. Temperature dependence of magnetic moment in a magnetic field of 100 Oe: non-magnetic $SrSi_2$ (red) and ferromagnetic $EuSi_2$ (blue) of equivalent thickness (≈2 nm).
